# Brain lipidomics and neurodevelopmental outcomes in intrauterine growth restricted piglets fed dairy or vegetable fat diets

**DOI:** 10.1038/s41598-022-07133-3

**Published:** 2022-02-28

**Authors:** Nicole L. Henriksen, Karina S. Asmussen, Xiaoyu Pan, Ping-Ping Jiang, Yuki Mori, Line I. Christiansen, Richard R. Sprenger, Christer S. Ejsing, Stanislava Pankratova, Thomas Thymann

**Affiliations:** 1 Section of Comparative Pediatrics and Nutrition, Department of Veterinary and Animal Sciences, University of Copenhagen, Dyrlægevej 68, 1870 Frederiksberg C, Denmark; 2 Center for Translational Neuromedicine, University of Copenhagen, Blegdamsvej 3B, 2200 Copenhagen N, Denmark; 3 Department of Biochemistry and Molecular Biology, VILLUM Center for Bioanalytical Sciences, University of Southern Denmark, Campusvej 55, 5230 Odense M, Denmark

**Keywords:** Developmental biology, Molecular biology, Neuroscience

## Abstract

Breast milk has neurodevelopmental advantages compared to infant formula, especially in low-birth-weight infants, which may in part relate to the fat source. This study compared neurodevelopmental outcomes in three-day-old normal birth weight (NBW) and intrauterine growth restricted (IUGR) piglets fed a formula diet with either vegetable oil (VEG) or bovine milk fat sources (MILK) for three weeks in a 2 × 2 factorial design. Behavioural tests, lipidomics, MRI and RNA sequencing analyses of plasma and brain tissue were conducted. The absolute levels of 82% and 11% of lipid molecules were different between dietary groups in plasma and hippocampus, respectively. Of the lipid molecules with differential abundance in the hippocampus, the majority were upregulated in MILK versus VEG, and they mainly belonged to the group of glycerophospholipids. Lower absolute brain weights, absolute grey and white matter volumes and behaviour and motor function scores, and higher relative total brain weights were present in IUGR compared to NBW with minor influence of diet. Cognitive function and cerebellar gene expression profiles were similar for dietary and weight groups, and overall only minor interactive effects between diet and birth weight were observed. Overall, we show that the dietary fat source influences the plasma and to a lesser degree the hippocampal lipidome and is unable to improve on IUGR-induced brain structural and functional impairments.

## Introduction

Breast milk is the preferred nutrition for infants^1^. However, when unavailable, infant formula constitutes an important source of nutrition^1^. The fat fraction of human breast milk is crucial in providing energy and nutrients to the developing infant brain^2^. Bovine milk fat used to be a common fat source in infant formula, however, today bovine milk fat has partially or completely been substituted with vegetable oils^3^. Vegetable oils contain higher levels of polyunsaturated fatty acids, but are generally less diverse in their lipid profile than human and bovine milk as most contain little to no cholesterol, sphingomyelin or short- and odd-chain fatty acids^4^. Contrary to vegetable oils, fat globules in human and bovine milk are emulsified by milk fat globule membranes, which are made up of a variety of bioactive lipids and proteins organised as a trilayer^5^. Additionally, structural differences in the arrangement of fatty acyl chains at the sn-positions of the triacylglycerol (TAG) molecules exist between fat sources, which may affect digestion and absorption^4,6^.

Beneficial effects of infant formula containing dairy lipids on the gut and brain have previously been reported. For example, emulsification of TAG globules with fractions enriched in bovine milk polar lipids improved in vitro TAG hydrolysis and in vivo absorption in neonatal piglets relative to soy lecithin^7,8^. Dairy fat blends enriched in α-linolenic acid have also been reported to increase brain docosahexaenoic acid accretion in juvenile rats compared to vegetable blends^9^. Finally, in clinical studies, supplementation of infant formula with bovine milk fat globule membranes has shown positive effects on language, cognitive and motor scores in term-born infants during the first year of life^10,11^. These effects may be even more prominent in intrauterine growth restricted (IUGR) infants, in which placental insufficiency of varying origin and compromised intestinal development have the potential to decrease nutrient uptake^12,13^. Indeed, IUGR infants have an increased risk of neurodevelopmental impairments during childhood^14^. In pigs, IUGR occurs spontaneously and has been suggested to be a good translational model of neurodevelopment in IUGR infants, as they show similar structural and functional changes to the brain such as compromised white matter development and learning deficits^15,16^.

Beyond the comparison of breastfeeding and infant formula^17^, the role of dietary fat source in neurodevelopment is not well studied. Here, we investigated whether changes in plasma lipids related to feeding diets with either vegetable or bovine fat prompted parallel changes to the hippocampal lipidome and whether this was beneficial for neurodevelopmental outcomes, especially in IUGR piglets.

## Materials and methods

### Study protocol

A total of 36 term-born piglets (Danish landrace × Yorkshire × Duroc) of both sexes and from six litters were selected from a commercial farm on the day of birth based on the following criteria: 18 normal birth weight (NBW, average: 1394 g) and 18 IUGR (< 1000 g, altered head morphology^18^). At 3 days of age (study day 1), piglets were transferred to an animal facility where they were stratified by sex and birth weight and randomized to receive a formula diet with either a vegetable oil (VEG) or bovine milk fat source (MILK) in a 2 × 2 factorial study design (n = 9 in each group). Behavioural tests (open field test, behavioural and motor function score, in-cage cognition test) were conducted between day 7–20. On either study day 21 or 22 (randomized), piglets were sedated with a combination of tiletamine-zolazepam (Zoletil 50 Vet, Virbac, Carros, France), xylazine (Xysol Vet, ScanVet, Fredensborg, Denmark), ketamine (Ketaminol Vet, MSD Animal Health, Copenhagen, Denmark) and butorphanol (Morphasol Vet, Orion Pharma, Espoo, Finland) and subsequently euthanized by intra-cardiac injection of pentobarbital (Euthanimal, 400 mg/ml, Scanvet). At necropsy, brain regional weights were recorded, and the right brain hemisphere, plasma, hippocampus and cerebellum samples were obtained for RNA sequencing (RNA-seq), lipidomics and magnetic resonance imaging (MRI) analysis. Animal experimental work was approved by the Danish Animal Experiments Inspectorate (license no.: 2014-15-0201-00418) and performed in accordance with Directive 2010/63/EU of the European Parliament and ARRIVE guidelines.

### Nutrition, housing and health

Piglets were fed formula diets which were designed to meet the nutritional requirements of piglets (Table 1). All piglets received controlled and equal amounts of formula every 3 h ranging from 160 to 280 ml/kg/day. Piglets were randomly assigned to and housed individually in cages equipped with a soft mat, an infrared heat source and cloth and ball for environmental enrichment. Body weight was recorded and clinical and feces scores were assigned daily^19^. On day 2, all piglets were treated prophylactically against coccidiosis with toltrazuril (Baycoxine Vet, 50 mg/ml, 0.4 ml/kg, Bayer Animal Health GmbH, Leverkusen, Germany). Piglets that developed diarrhea were additionally treated with gentamicin (Gentocin, Vet, 4.35 mg/ml, 5 mg/piglet, ScanVet) and in ongoing cases, zinc oxide (Urtegaarden, Allingåbro, Denmark) and electrolytes (Revolyt, Gunnar Kjems, Copenhagen, Denmark). Three piglets were lame at arrival and were treated with meloxicam (Metacam, 5 mg/ml, 0.08 ml/kg, Boehringer Ingelheim, Ingelheim am Rhein, Germany) and enrofloxacin (Baytril Vet, 50 mg/ml, 2.5 mg/kg, Bayer Animal Health GmbH) for 1–3 days.

**Table 1:** Composition of the vegetable oil (VEG) and bovine milk fat (MILK) based diets.

Constituents	Product	Unit	VEG	MILK
Whey protein	WPI/WPC 90^a^	g/L	30	30
Casein	Miprodan 40^a^	g/L	30	30
Lactose, minerals	Variolac 855^a^	g/L	70	70
Vitamins, minerals	Phlexy-Vits^b^	g/L	5	5
Fresh bovine cream	Cream 38%^c^	g/L	0	119
Vegetable oils (long-chained triacylglycerides)	Liquigen^b^	g/L	40	0
Vegetable oils (medium-chained triacylglycerides)	Calogen^b^	g/L	50	0
Energy		kJ/L	3614	3731
Protein		g/L	55	58
Carbohydrate		g/L	60	62
Fat		g/L	45	46
Saturated fat		g/L	26	30
Monounsaturated fat		g/L	12	12
Polyunsaturated fat		g/L	6	1

^a^Arla Food Ingredients, Viby J, Denmark.

^b^Nutricia, Amsterdam, The Netherlands.

^c^Arla Foods amba, Viby J, Denmark.

### Behaviour, cognition and motor function

On day 7, 13 and 20, an open field test evaluating exploration and locomotor activity was conducted. Piglets were placed in a 2 × 2 m arena and recorded for 4 min. The Ethovision software (Version 10, Noldus, Wageningen, The Netherlands) was used to generate measurements of velocity, distance travelled, and time spent in central, peripheral and corner zones of the arena, and the number of rearings were recorded. Zones of the open field arena are shown in Supplementary Fig. S1. On day 7, piglets were assigned a behavioural and motor score during the open field session by two independent observers. The motor score consisted of the following parameters that were scored on a scale of 1–3 every 30 s for 1.5 min and tallied to provide a total score of maximum 36 for each piglet: activity (1: resting, 2: intermediate, 3: active), gait (1: stiff, 2: intermediate, 3: free), posture (1: hunched, 2: intermediate, 3: upright), head position (1: tilted, 2: turned, 3: neutral). The behavioural score was conducted in the same way evaluating exploration (1: resting, 2: intermediate, 3: active) and anxiety (1: fearful, 2: nervous, 3: calm) with a maximum score of 18. Thus, lower motor and behavioural scores indicated more compromised motor function and anxious piglets, respectively. Day 7 tests were postponed to day 9 in five piglets (equally distributed between groups) due to severe diarrhea, and these were included with the other day 7 data. Observers were blinded to dietary groups but due to obvious differences in size it was not possible to be blinded to weight groups.

An in-cage cognition test (NorthTech Aps, Copenhagen, Denmark) was conducted 1–4 times a day from day 9 to 20. During each 1-h test period, one of two light cues situated on the inside of each cage door was turned on, and piglets had to approximate the light cue to gain a 1 ml apple juice reward. If piglets approximated the wrong light cue, a 60 s penalty would be elicited where lights would be turned off. This task was repeated 10 times during each test period with the position of the light cue alternating randomly. The percentage of correct responses per day were registered. Piglets that answered ≤ 20% in every trial throughout the study were excluded.

### Lipidomics

Frozen hippocampal tissue samples (average: 330 mg) were homogenized in 750 µl 80 °C 155 mM ammonium formate buffer using an ULTRA-TURRAX (IKA, Staufen, Germany), followed by total protein determination using the Pierce BCA Protein Assay Kit (Thermo Fisher Scientific, Waltham, Massachusetts, USA). For lipidomics analysis, the equivalent of 10 µg total protein was used for hippocampus samples and 7 µl for plasma samples. In both cases, the samples were spiked with internal lipid standards, followed by a two-step lipid extraction as previously described^20,21^. Lipid extracts were analyzed by high-resolution MS^ALL^ lipidomics using an Orbitrap Fusion Tribrid (Thermo Fisher Scientific) equipped with a robotic TriVersa NanoMate ion source (Advion Biosciences, Ithaca, New York, USA), as described previously^21,22^. Briefly, as described by Sprenger et al.^21^, apolar 10:1 lipid extracts were loaded in 96-well plates and mixed with 13.3 mM ammonium formate in 2-propanol for positive ion mode analysis or with 1.33 mM ammonium formate in 2-propanol for negative ion mode analysis. Polar 2:1 lipid extracts were mixed with 0.01% methylamine in methanol and analyzed in negative ion mode. Samples were infused using a back pressure of 1.25 psi and ionization voltage of ± 0.95 kV. FTMS data were recorded using a maximal injection time of 0.1 s, automated gain control at 100000, 3 microscans and a target resolution of 500.000. FTMS2 data were acquired using a maximal injection time of 0.1 s, automated gain control at 50000, 3 microscans and target resolution of 30.000, and all FTMS data were recorded at an ion transfer tube temperature of 275 °C^21^. Lipid identification was performed using the ALEX^123^ software suite^23,24^, and quantification of lipid molecules as well as in silico total fatty acyl analysis were done by data processing pipelines in SAS 9.4 (SAS Institute Inc., Cary, North Carolina, USA)^25^. Lipid class abbreviations are shown in the Fig. 2 legend.

### MRI

The right brain hemisphere from seven randomly selected piglets from each group was fixed in 4% paraformaldehyde. In preparation for post-mortem imaging, brains were placed in an imaging tube filled with Fomblin (perfluoro-polyether; Solvay, Princeton, New Jersey, USA) to reduce susceptibility artefacts and avoid dehydration. All data were acquired from a 9.4 Tesla preclinical scanner (Bruker BioSpin, Ettlingen, Germany) equipped with a 240 mT/m gradient coil (BGA-12S, Bruker). Images were acquired using an 86-mm inner diameter transmit-receive volume coil. The imaging protocol consisted of a 3D gradient-spoiled steady-state free precession (3D-FISP) sequence to discriminate the contrast between white matter and grey matter, and imaging parameters were: repetition time = 4.6 ms, echo time = 2.3 ms, number of signals averaged = 10, flip angle = 25°, field of view = 60 mm × 38.4 mm × 25.6 mm, matrix = 300 × 192 × 128, image resolution = 200 µm isotropic, acquisition time = 20 min. The image bias field was removed using Advanced Normalization Tools (ANTs N4 bias correction)^26^. The total brain volume of each sample was automatically segmented by using region growing with ITK-snap (version 3.8.0, www.itksnap.org)^27^. Moreover, pixel intensity factorized semi-automatic thresholding was conducted to segment the white matter structure. The volume of the whole brain, white matter and grey matter were measured, and the volume of white and grey matter were normalized to the total volume of the brain hemisphere (Fig. 4A).

### RNA-seq

Snap-frozen cerebellum tissue was crushed, homogenized in QIAzol lysis reagent (Qiagen, Hilden, Germany), and total RNA was extracted using the RNeasy Li pid Tissue Mini Kit (Qiagen). Bioanal yzer 2100 (Agilent, Santa Clara, California, USA) derived RNA integrity numbers ranged from 6.50 to 8.80 with the exception of three samples (1 VEG-IUGR, 1 VEG-NBW, 1 MILK-NBW) that were excluded due to values below 5.00. Sequencing libraries were constructed by Novogene Europe (Cambridge, UK), sequenced using the Illumina NovaSeq platform (Illumina, San Diego, California, USA), and subsequent data pre-processing and analysis were conducted as previously described^28^.

### Statistics

Statistics were conducted in R version 4.0.3 (R Foundation for Statistical Computing, Vienna, Austria). Continuous data were analysed by a linear mixed model with sex as a fixed effect, litter as a random effect and an interaction between diet and weight. For longitudinal data, the model additionally included piglet as a random effect and a diet × weight × day interaction. Count data were analysed by a negative binomial generalized linear model using DESeq2. Unsupervised principal component analysis using the pcaMethods package was applied to autoscaled molecular lipid species data. Normality and homoscedasticity assumptions were checked using quantile–quantile and residual vs. fitted plots and data were transformed when required. A logarithmic transformation was used for lipidomics data to approximate a normal distribution. Data that were not normally distributed were analysed by a non-parametric Mann–Whitney test. A Benjamini–Hochberg multiple testing correction was used for RNA-seq and class, molecular species and in silico lipidomics data. Probability values below 0.05 were considered statistically significant. If no interactions were present, data were pooled and presented as main effects of either diet or birth weight (mean ± standard deviation).

## Results

### Clinical outcomes

Growth data from these piglets has previously been published^29^ and showed that bodyweight was lower in IUGR compared to NBW piglets throughout the study, whereas there were no differences between dietary groups, and neither diet nor birth weight affected growth rates^29^. All piglets were scored as clinically healthy (score 1–2) throughout the study, and the diarrhea incidence was similar between groups (VEG: 78%, MILK: 78%; NBW: 72%, IUGR: 83%).

### Behaviour, cognition and motor function

**Figure 1:**
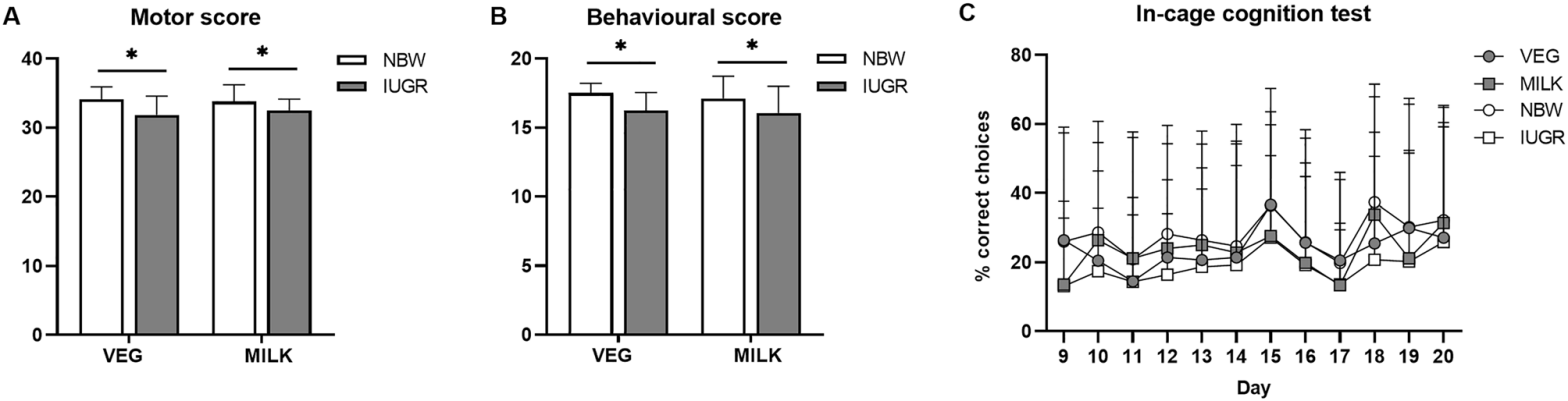
Behavioural, cognitive and motor function tests. (**A**) Motor score day 7, (**B**) behavioural score day 7 and (**C**) in-cage cognition test—percentage correct choices on day 9–20. In (**A**) and (**B**), n = 9. In (**C**), data are pooled and presented as main effects of diet and birth weight (n = 18). Data are shown as mean ± SD. All data were analysed using a linear mixed model. Statistically significant differences between groups are shown as *p < 0.05. All figures were created with GraphPad Prism (version 9.3.0, GraphPad Software, San Diego, California, USA). *VEG* vegetable oil, *MILK* bovine milk fat, *NBW* normal birth weight, *IUGR* intrauterine growth restricted.

NBW piglets received higher behavioural and motor scores (Fig. 1A,B) and spent more time in zone 3 of the open field arena compared to IUGR piglets on day 7 (Table 2). There were no effects of diet or birth weight, nor any interactions between them for any of the remaining open field parameters (Table 2). In the in-cage cognition test, eight piglets (2 VEG-NBW, 2 VEG-IUGR, 1 MILK-NBW, 3 MILK-IUGR) were excluded based on predetermined criteria outlined in the method section. The percentage of correct responses in this test was similar across groups (Fig. 1C). There were no effects of sex in any of the tests.

**Table 2:** Open field parameters on day 7, 13 and 20. Data are pooled and presented as main effects of diet and birth weight (n = 18). Data are shown as mean ± SD. All data were analysed using a linear mixed model and p < 0.05 is considered statistically significant. *VEG* vegetable oil, *MILK* bovine milk fat, *NBW* normal birth weight, *IUGR* intrauterine growth restricted.

Parameter^a^		Day 7				Day 13				Day 20				p value^b^	
		VEG	MILK	NORM	IUGR	VEG	MILK	NORM	IUGR	VEG	MILK	NORM	IUGR	p_DIET_	p_WEIGHT_
Distance	m	27.1 ± 11.0	26.5 ± 11.0	29.5 ± 10.8	24.1 ± 10.6	37.0 ± 13.3	35.3 ± 13.1	40.0 ± 15.0	32.3 ± 9.65	36.2 ± 9.67	36.7 ± 17.3	38.9 ± 16.1	34.0 ± 10.9	0.8	0.09
Velocity	cm/s	11.3 ± 4.59	11.1 ± 4.60	12.3 ± 4.49	10.1 ± 4.40	15.4 ± 5.56	14.7 ± 5.44	16.7 ± 6.24	13.4 ± 4.02	15.1 ± 4.02	15.3 ± 7.20	16.2 ± 6.73	14.2 ± 4.54	0.8	0.09
Zone 1	s	14.0 ± 18.2	7.41 ± 9.26	9.51 ± 10.5	11.9 ± 18.1	14.4 ± 15.7	10.9 ± 10.5	15.2 ± 14.4	10.1 ± 12.0	9.42 ± 11.0	11.9 ± 8.28	7.16 ± 4.88	14.1 ± 12.0	0.7	0.9
Zone 2	s	41.1 ± 37.2	38.5 ± 24.1	47.6 ± 22.0	31.9 ± 36.8	42.2 ± 21.7	35.8 ± 22.9	44.8 ± 24.4	33.3 ± 18.8	32.2 ± 25.3	25.7 ± 24.6	33.6 ± 26.6	24.3 ± 22.8	0.5	0.06
Zone 3	s	8.93 ± 11.8	10.0 ± 11.4	14.1 ± 13.5	4.84 ± 6.53	13.9 ± 16.0	16.8 ± 15.7	15.7 ± 15.7	15.0 ± 16.2	7.24 ± 8.03	6.48 ± 7.67	5.84 ± 6.78	7.88 ± 8.69	0.3	#
Zone 4	s	6.18 ± 11.6	6.82 ± 9.38	6.82 ± 9.87	6.18 ± 11.2	9.60 ± 12.7	6.95 ± 8.84	6.97 ± 8.14	9.57 ± 13.2	4.97 ± 5.79	9.14 ± 15.1	3.38 ± 4.54	10.7 ± 14.9	0.8	0.5
Center	s	72.8 ± 49.8	69.5 ± 39.2	59.7 ± 39.2	82.7 ± 47.0	54.1 ± 26.6	68.8 ± 47.7	60.1 ± 44.1	62.7 ± 33.9	80.5 ± 34.5	88.9 ± 49.3	89.4 ± 47.5	80.1 ± 36.9	0.6	0.7
Periphery	s	167 ± 49.8	171 ± 39.2	180 ± 39.1	157 ± 47.0	186 ± 26.6	171 ± 47.6	180 ± 44.0	177 ± 33.9	160 ± 34.5	151 ± 49.3	151 ± 47.5	160 ± 36.9	0.2	0.8
Rearings		0.56 ± 1.46	1.11 ± 2.56	0.94 ± 2.26	0.72 ± 1.93	3.28 ± 3.55	3.00 ± 3.58	3.06 ± 2.75	3.22 ± 4.22	1.11 ± 1.41	0.78 ± 1.93	1.00 ± 1.85	0.89 ± 1.53	0.5	0.9

^#^A weight × day interaction for time spent in zone 3 of the arena is present (day 7: p < 0.01, day 13: p = 0.8, day 20: p = 0.6).

^a^Supplementary Fig. S1 defines each zone of the open field arena.

^b^The overall p value indicating differences between either dietary or weight groups over time.

### Brain regional weights and cerebral water fraction

Main effects of birth weight were present for absolute and relative brain weights with absolute total, cerebellum, cerebrum, brain stem and hippocampus weights being higher (all p < 0.05) and relative total weight (p < 0.001) being lower in the NBW compared to IUGR group (Table 3). There were also main effects of diet in that the MILK group had higher relative cerebellum and brain stem weights and lower relative cerebrum weights than the VEG group (all p < 0.05) (Table 3). The cerebral water fraction was similar across groups (Table 3). There were no interactions between diet and birth weight.

**Table 3:** Absolute and relative regional brain weights. Data are pooled and presented as main effects of diet and birth weight (n = 18). Data are shown as mean ± SD. All data were analysed using a linear mixed model except water percentage which was analysed by a Mann–Whitney test. p < 0.05 is considered statistically significant. *VEG* vegetable oil, *MILK* bovine milk fat, *NBW* normal birth weight, *IUGR* intrauterine growth restricted.

	Diet		Birth weight		p value	
	VEG	MILK	NBW	IUGR	p_DIET_	p_WEIGHT_
**Absolute brain weights (g)**						
Total brain	46.9 ± 3.33	46.1 ± 3.43	48.7 ± 2.57	44.3 ± 2.50	0.3	< 0.001
Cerebellum	5.03 ± 0.36	5.11 ± 0.42	5.27 ± 0.33	4.87 ± 0.33	0.7	< 0.01
Cerebrum	36.4 ± 2.69	35.6 ± 2.84	37.9 ± 2.17	34.2 ± 1.91	0.3	< 0.001
Brain stem	4.70 ± 0.40	4.85 ± 0.46	4.94 ± 0.32	4.62 ± 0.48	0.3	< 0.05
Left hippocampus	0.77 ± 0.10	0.74 ± 0.09	0.80 ± 0.08	0.71 ± 0.08	0.3	< 0.01
Caudate nucleus	0.39 ± 0.07	0.41 ± 0.05	0.42 ± 0.06	0.39 ± 0.06	0.4	0.1
Water percentage (%)^a^	0.818 ± 0.004	0.818 ± 0.004^b^	0.819 ± 0.003	0.817 ± 0.005^b^	0.8	0.2
**Relative brain weights (%)**^c^						
Total brain	1.42 ± 0.40	1.33 ± 0.39	1.21 ± 0.18	1.63 ± 0.38	0.7	< 0.001
Cerebellum	10.7 ± 0.49	11.1 ± 0.33	10.8 ± 0.47	11.02 ± 0.41	< 0.05	0.06
Cerebrum	77.7 ± 0.86	76.9 ± 0.90	77.6 ± 1.00	77.0 ± 0.85	< 0.05	0.05
Brain stem	10.0 ± 0.47	10.5 ± 0.70	10.1 ± 0.50	10.4 ± 0.74	< 0.05	0.2
Left hippocampus	1.64 ± 0.17	1.61 ± 0.13	1.65 ± 0.15	1.60 ± 0.16	0.6	0.2
Caudate nucleus	0.84 ± 0.14	0.90 ± 0.13	0.86 ± 0.13	0.88 ± 0.14	0.2	0.8

^a^Water percentage = left cerebral hemisphere weight before and after dehydration for 2 weeks at 50 °C.

^b^One piglet was removed due to a measurement error (n = 17).

^c^Regional brain weights as a percentage of total brain weight or total brain weight as a percentage of body weight.

### Lipidomics

**Figure 2:**
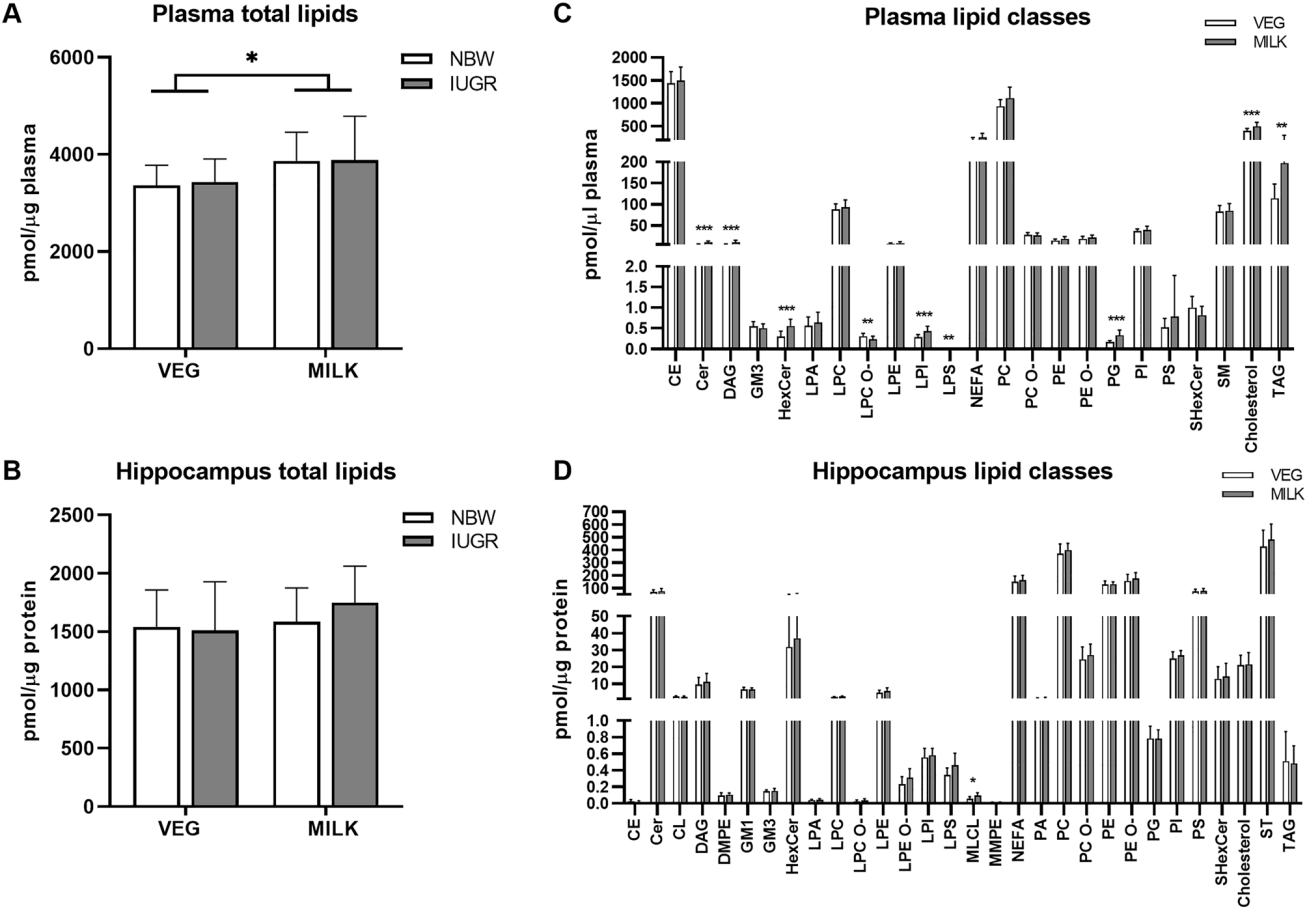
Total lipids (**A**,**B**) and lipid class abundances (**C**,**D**) in plasma and hippocampus on day 21–22. In (**A**) and (**B**), n = 9. In (**C**) and (**D**), data are pooled and presented as main effects of diet (n = 16–18). Data are shown as mean ± SD. All data were analysed using a linear mixed model. A Benjamini–Hochberg correction was applied in (**C**) and (**D**). Statistically significant differences between groups are shown as *p < 0.05, **p < 0.01 and ***p < 0.001. All figures were created with GraphPad Prism (version 9.3.0, GraphPad Software, San Diego, California, USA). *VEG* vegetable oil, *MILK* bovine milk fat, *NBW* normal birth weight, *IUGR* intrauterine growth restricted, *CE* cholesteryl ester, *Cer* ceramide, *CL* cardiolipin, *DAG* diacylglycerol, *DMPE* dimethyl-phosphatidylethanolamine, *GM1* monosialotetrahexosylganglioside, *GM3* monosialodihexosylganglioside, *HexCer* hexosyl ceramide, *LPA* lysophosphatidic acid, *LPC* lysophosphatidylcholine, *LPC O-* lysoalkylphosphatidylcholine, *LPE* lysophosphatidylethanolamine, *LPE O-* lysoalkylphosphatidylethanolamine, *LPI* lysophosphatidylinositol, *LPS* lysophosphatidylserine, *MLCL* monolysocardiolipin, *MMPE* monomethyl-phosphatidylethanolamine, *NEFA* non-esterified fatty acid, *PA* phosphatidic acid, *PC* phosphatidylcholine, *PC O-* alkylphosphatidylcholine, *PE* phosphatidylethanolamine, *PE O-* alkylphosphatidylethanolamine, *PG* phosphatidylglycerol, *PI* phosphatidylinositol, *PS* phosphatidylserine, *SHexCer* Sulfatide hexosyl ceramide, *SM* sphingomyelin, *ST* cholesterol, *TAG* triacylglycerol.

A total of 863 molecular lipid species were identified in plasma and 897 in the hippocampus encompassing 23 and 30 lipid classes, respectively. The total concentration of lipids in plasma was higher in the MILK compared to VEG group, whereas it was similar between dietary groups in the hippocampus (Fig. 2A,B). Figure 2C,D depict the diet-induced changes in plasma and hippocampal lipid classes. In plasma, the MILK diet increased the molar concentrations of Cer by 86%, DAG by 93%, HexCer by 84%, LPI by 51%, LPS by 134%, PG by 101%, cholesterol by 24% and TAG by 73% and decreased the concentration of LPC O-by 25% compared to the VEG diet (all p < 0.01). MLCL, which was 62% higher in the MILK compared to VEG group, was the only change in the hippocampus (p < 0.05).

**Figure 3:**
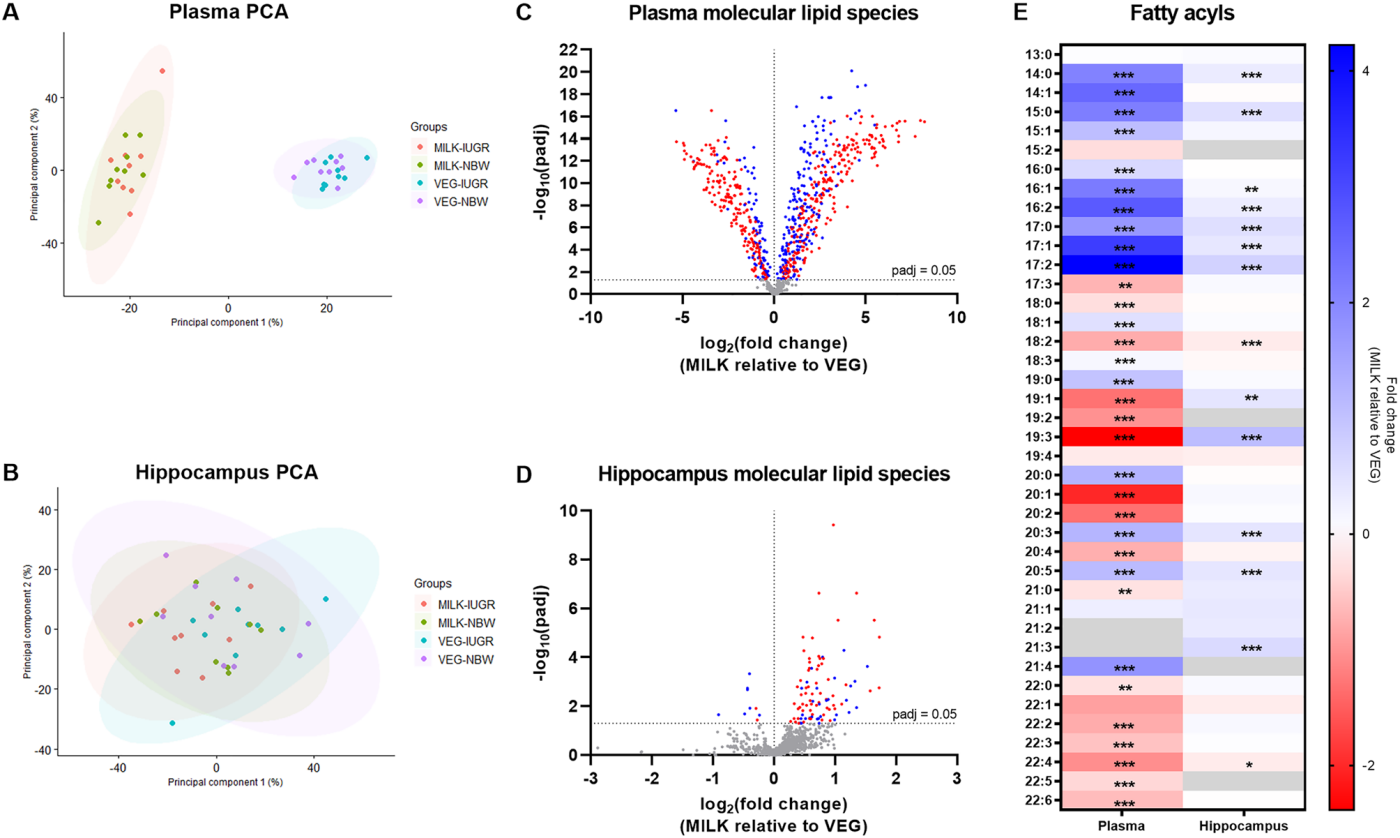
Plasma and hippocampal lipidomics on day 21–22. (**A**,**B**) Principal component analysis plots of absolute molecular lipid species data, (**C**,**D**) volcano plots of molecular lipid species in plasma and hippocampal tissue, (**E**) total fatty acyl analysis. In (**C**), red dots indicate triacylglycerol species. In (**D**), red dots indicate glycerophospholipid species. Grey cells in (**E**) indicate that the fatty acyl chain was b elow the lower limit of detection. In (**A**) and (**B**), n = 9. In (**C**,**D**) and (**E**), data are pooled and presented as main effects of diet (n = 12–18). Data are shown as fold-change of the MILK relative to VEG group (**C**,**D**,**E**). Data were analysed using a principal component analysis (**A**,**B**) or linear mixed model (**C**,**D**,**E**). A Benjamini–Hochberg correction was applied in (**C**), (**D**) and (**E**). Statistically significant differences between groups are shown as *p < 0.05, **p < 0.01, ***p < 0.001 or as values above the horizontal dotted line (padj < 0.05). Figures were created with R (version 4.0.3, R Foundation for Statistical Computing, Vienna, Austria) (**A**,**B**) and GraphPad Prism (version 9.3.0, GraphPad Software, San Diego, California, USA) (**C**,**D**,**E**). *VEG* vegetable oil, *MILK* bovine milk fat, *NBW* normal birth weight, *IUGR* intrauterine growth restricted.

Principal component analysis of molecular lipid species data demonstrated a separation between dietary groups in plasma but not the hippocampus (Fig. 3A,B). In plasma, the molar abundance of 704 lipid molecules (82% of total) differed between the dietary groups and of the differentially abundant lipids, TAG species dominated (60%) (Fig. 3C). These TAG molecules predominantly featured longer acyl lengths in the MILK (C54–58) compared to VEG (C48–54) group. In the hippocampus lipidome, 102 lipid molecules (11% of total) were differentially abundant between the dietary groups, with the majority being elevated in the MILK group and mainly consisting of glycerophospholipids (Fig. 3D). In silico total fatty acyl analysis of plasma revealed 16 fatty acyl chains that were more abundant in the VEG group and 17 in the MILK group (all p < 0.01). Here, the lipidome of piglets fed the MILK diet tended to have more saturated, monounsaturated and shorter fatty acyl chains compared to the VEG diet. In the hippocampus, the VEG diet resulted in higher abundance of lipids with 18:2 and 22:4 chains, whilst the MILK diet increased the abundance of 14:0, 15:0, 16:1, 16:2, 17:0, 17:1, 17:2, 19:1, 19:3, 20:3, 20:5 and 21:3 fatty acyls (all p < 0.05) (Fig. 3E). Among the significantly different lipid molecules, 25 molecular lipid species and 11 fatty acyls were similarly modulated in the plasma and hippocampus following the dietary interventions (Supplementary Fig. S2).

Statistical analysis demonstrated no main effects of birth weight at the lipid class-, molecular species- or total fatty acyl-level in neither plasma or the hippocampus. However, there were interactions between diet and birth weight for the total level of 19:2 and 22:5 fatty acyl chains in the hippocampus. A higher abundance of 19:2 was found in the MILK-IUGR compared to MILK-NBW, VEG-IUGR and VEG-NORM groups (all p < 0.05), and a lower abundance of 22:5 was found in VEG-NBW compared to MILK-IUGR, VEG-IUGR and MILK-NBW (all p < 0.001).

### MRI

**Figure 4:**
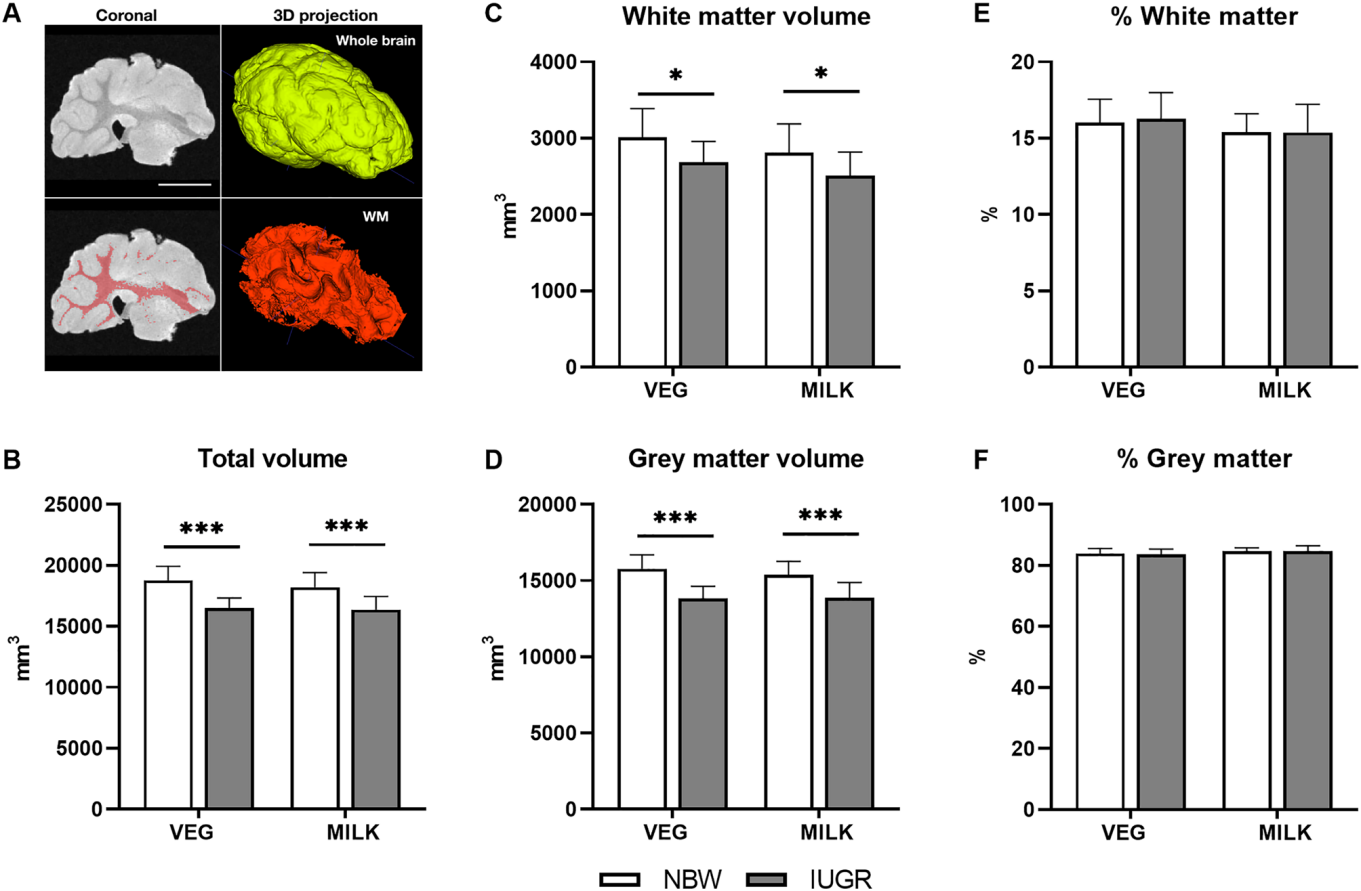
Magnetic resonance imaging on day 21–22. (**A**) A representative coronal and 3D reconstructed image showing the mapping of white matter (red), (**B**) absolute total volume, (**C**) absolute white matter volume, (**D**) absolute grey matter volume, (**E**) percentage white matter and (**F**) percentage grey matter of the right hemisphere of brains (n = 7). Scale bar = 1 cm. Data are shown as mean ± SD. All data were analysed using a linear mixed model. Statistically significant differences between groups are shown as *p < 0.05 and ***p < 0.001. Figures were created with ITK-SNAP (version 3.8.0, www.itksnap.org) (**A**) and GraphPad Prism (version 9.3.0, GraphPad Software, San Diego, California, USA) (**B**–**F**). *VEG* vegetable oil, *MILK* bovine milk fat, *NBW* normal birth weight, *IUGR* intrauterine growth restricted.

Absolute total brain, grey and white matter volumes were lower in IUGR compared to NBW piglets (all p < 0.05) (Fig. 4B–D), but the percentage of grey and white matter did not differ between groups (Fig. 4E,F). There were no effects of diet or interactions between diet and birth weight.

### RNA-seq

RNA-seq of the cerebellum showed no main effects of diet or birth weight, but interactions were present for four genes. Enhanced expression of Checkpoint Kinase 2 (CHEK2) and TP53RK-binding protein (TPRKB) were found in VEG-NBW compared to MILK-NBW piglets (both p < 0.05). Higher levels of Solute Carrier Family 19 Member 3 (SLC19A3) and lower levels of phospholipase A2 group IIE (PLA2G2E) were found in MILK-IUGR compared to MILK-NBW piglets, respectively (both p < 0.01) (Supplementary Fig. S3).

## Discussion

This study investigated how neurodevelopment in NBW and IUGR piglets was affected by feeding a diet with either a VEG or MILK source. These results demonstrate that absolute brain weights, absolute grey and white matter volumes and behaviour and motor function were compromised in IUGR piglets, whilst 82% of the plasma lipidome and 11% of the hippocampal lipidome and some relative regional brain weights were affected by diet with few interactive effects. The results suggest similar cerebellar gene expression profiles and cognitive function across the groups.

The developing brain has an increased sensitivity to dietary changes in the perinatal period^30^. In the present study we found that changes in the hippocampal lipidome were related to diet and not birth weight with only minor interactive effects indicating that IUGR infants do not benefit more from a particular dietary fat source in brain lipid accretion. Relative to VEG, the MILK diet showed a higher total amount of plasma lipids, which is in agreement with a previous study demonstrating increased fat absorption in infants fed human milk compared to formula^31^. Although substantial differences in the plasma lipidome were present between dietary groups, which is not surprising given the differences between fat sources^4^, significantly less changes occurred in the hippocampus suggesting that the brain lipidome is tightly controlled across all conditions. Differentially abundant lipid molecules in the hippocampus were largely independent of the plasma lipid profile. These results are similar to findings from piglets fed formula diets with different vegetable oil blends or sow milk, which demonstrated no correlation between the composition of major fatty acyl chains of plasma and brain phospholipids^32^. In a study of young rats fed infant formula or human milk, compositional differences in the fat fraction of the diets also did not result in corresponding changes in the brain with the concentration of SM being the only observed difference in the brain, which was unrelated to its concentration in the diets^33^. This may in part be explained by the fact that metabolic remodelling of dietary lipids to free fatty acids is important for transport across the blood–brain barrier^34^ and that in addition to diet, body fat stores and local synthesis of lipids are sources of brain lipids^33–35^. Additionally, studies of the fetal and infant brain show that concentrations of different lipids peak at different stages of development, and the brain may therefore only acquire what it needs^36^. Ultimately, we found that the MILK diet relative to VEG resulted in larger accretion of specific lipids in the hippocampus. The MILK diet mainly increased the MLCL lipid class and several phospholipid species in the hippocampus. Dietary phospholipids may influence cognitive function by several mechanisms including providing substrate for acetylcholine synthesis for cholinergic neurotransmission, regulating glutamate receptor density and functioning as precursors for secondary messengers in important intracellular signalling pathways^37^.

In line with previous reports on IUGR piglets^15,38^, this study demonstrated reductions in absolute but not relative grey and white matter volumes by MRI and absolute total and regional brain weights in t he IUGR compared to NBW group. Additionally, the increased relative total brain weights of IUGR piglets could reflect brain-sparing, which despite being considered a protective mechanism^39^ has been associated with a higher risk of neurodevelopmental impairments^14^. Feeding the MILK diet was associated with an increase in the relative cerebellum and brain stem weights and a decrease in the relative cerebrum weight compared to the VEG diet. Modifications to the dietary fat fraction in early life, including hydrolysed fat and phospholipid enrichment, have been shown to influence regional brain, grey and white matter volumes in 26–29 day-old piglets^38,40^. However, diet-induced changes to relative brain weights in this study were not reflected in the MRI data as volumes were similar between dietary groups. This may have differed had these parameters been studied at a regional level. Unlike a previous report describing an influence of hydrolysed dietary fat on reduction of the hippocampal expression of immune response-related genes in small and appropriate for gestational aged piglets^38^, the present study only showed minor interactive effects between diet and birth weight in the cerebellar transcriptome, which were unrelated to the immune system. Surprisingly, we were also unable to demonstrate the broad brain transcriptome differences that have recently been reported between 14 day-old small and appropriate for gestational aged piglets^38^. This could in part relate to differences in the analysed brain region and older age of piglets in the present study.

A causal relationship between dietary lipids, the brain lipidome and neurological function has previously been suggested^41^. Therefore, this study also evaluated behaviour, cognition and motor function. While IUGR piglets performed worse than NBW piglets in behavioural and motor functional tests on day 7, we found no influence of diet, nor any effects of diet or birth weight on cognitive function from day 9–20. Hence, the smaller diet-related changes to the brain lipidome did not influence neurodevelopmental outcomes. The lack of differences in cognitive function between weight groups could be explained by the cognitive test used. Development of the in-cage cognition test is an effort to replace labour intensive cognitive tests for pigs by mimicking simplified touchscreen-based associative tests used in other species^42^. Current limitations of this test are, however, that the number of participating animals in their home cages varies between trials resulting in lower sample sizes in this study, and it may with the current setup not be sensitive enough to detect smaller differences in cognitive function. As this test is not currently routinely applied in piglets, this data should therefore be confirmed using more verified cognitive tests such as the T-maze^43,44^.

The present study utilized fat sources of different origin. It is important to note that there are advantages to both bovine and vegetable fat sources^4^, and that a combination may be more effective in resembling human breast milk. The optimal fat source, composition and dosage, and timing of dietary interventions to enhance neurodevelopment require further research, where animal models with high translational value are essential for understanding how nutrition impacts the brain in early life^45^.

## Conclusion

In summary, we report that dietary lipids of vegetable or bovine origin provide markedly different plasma lipid compositions, but the majority of these differences do not manifest in the hippocampal lipidome. Furthermore, we found that the dietary fat source had limited impacts on IUGR-induced structural and functional changes to the brain of neonatal piglets. A further understanding of the interplay between diet, and specific organ lipid metabolism in early life seems key in determining how diet can affect neurodevelopment.

## Supplementary Information

Supplementary Figures.

## Data Availability

Datasets can be acquired from the corresponding author upon reasonable request. Transcriptome data have been deposited in the NCBI Gene Expression Omnibus database (Accession number GSE182263).
