# Supplementary material for: Brain lipidomics and neurodevelopmental outcomes in intrauterine growth restricted piglets fed dairy or vegetable fat diets
Source: Sci Rep. 2022 Feb 28;12:3303. doi: 10.1038/s41598-022-07133-3 (PMC8885751; doi:10.1038/s41598-022-07133-3)
Supplement: Supplementary file 1 — Supplementary Figures. [file 41598_2022_7133_MOESM1_ESM.pdf]

Supplementary Figure S1

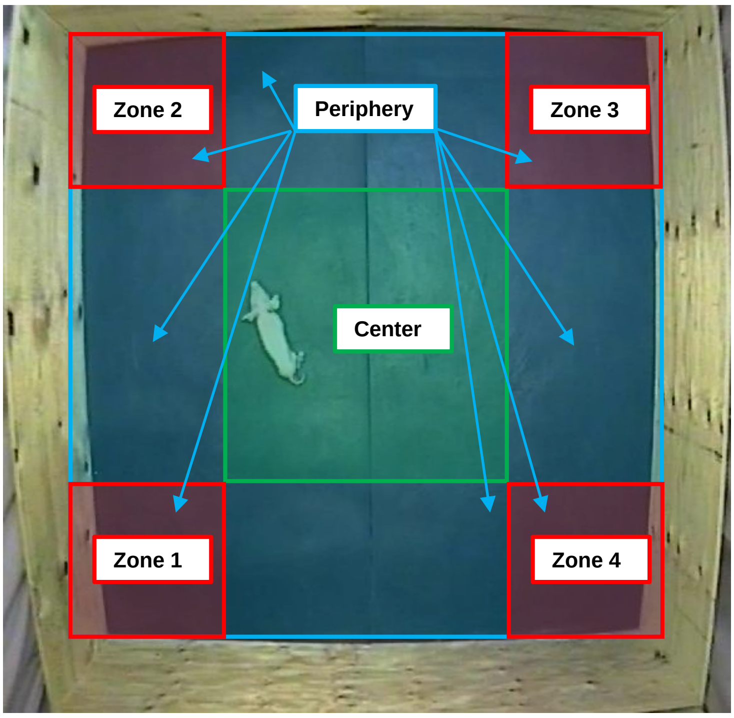

Supplementary Figure S1: Zones of the open field arena.

Supplementary Figure S2

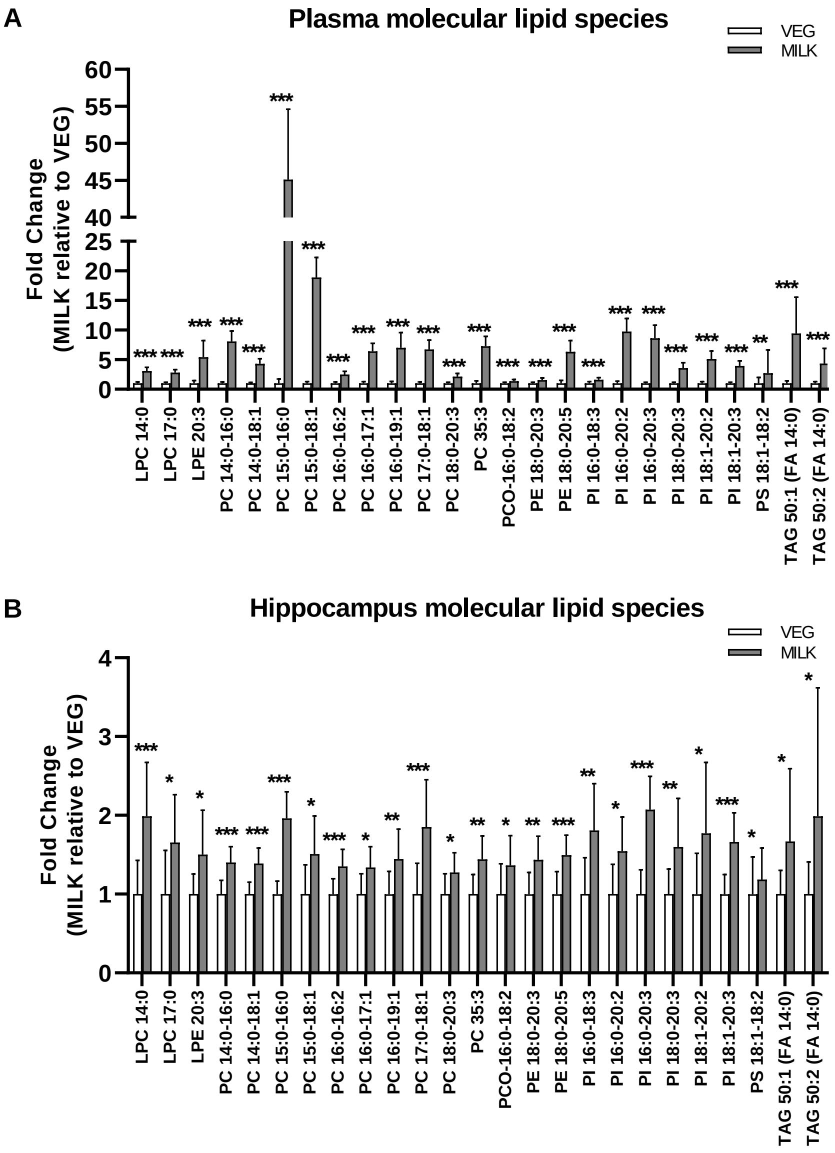

Supplementary Figure S2: Differentially abundant molecular lipid species that are in agreement between plasma (A) and the hippocampus (B) in piglets fed vegetable oil (VEG) or bovine milk fat (MILK) diets. Data are pooled and presented as main effects of diet (n=14-18). Data are shown as fold-change of the MILK relative to VEG group. Statistical analysis was conducted on the molar data. All data were analysed using a linear mixed model and a Benjamini-Hochberg correction was applied. Statistically significant differences between groups are shown as *$p<0.05$, **$p<0.01$, ***$p<0.001$. All figures were created with GraphPad Prism (version 9.3.0, GraphPad Software, San Diego, California, USA).

Supplementary Figure S3

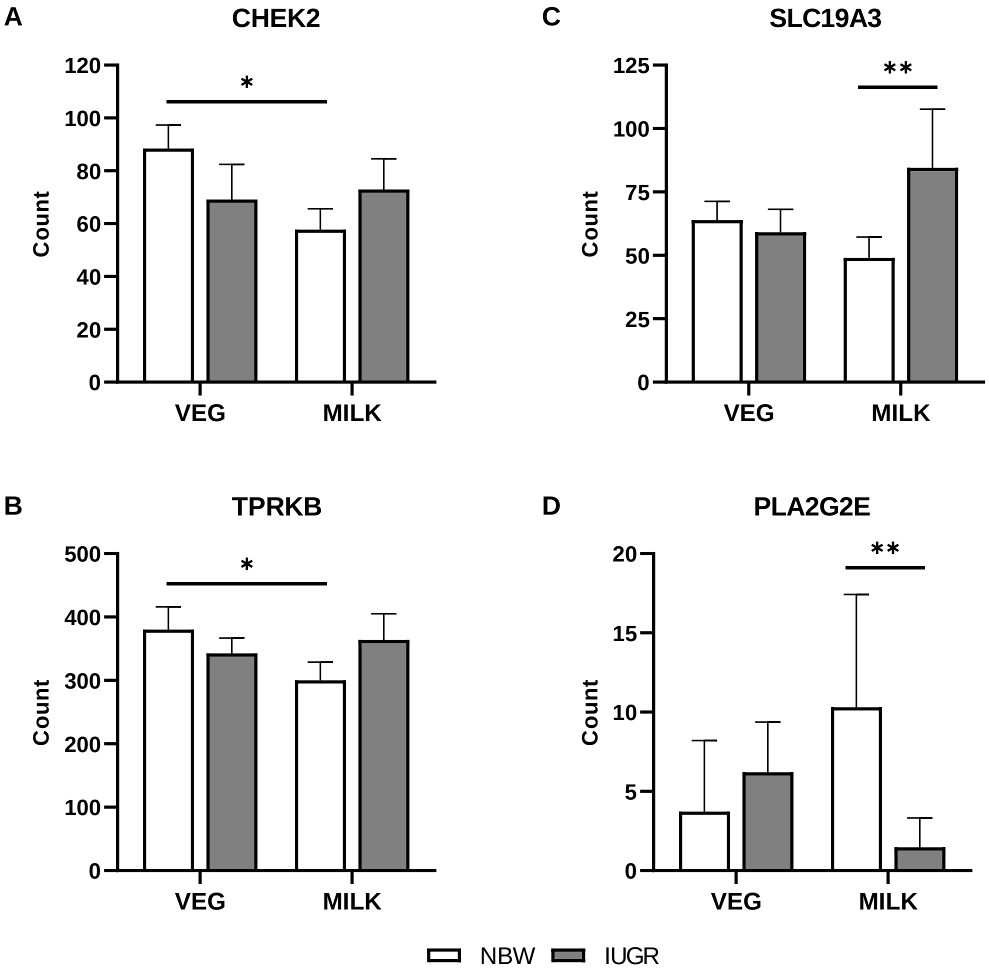

Supplementary Figure S3: Cerebellar gene expression on day 21-22 (n=8-9). Data are shown as mean±SD. All data were analysed using a negative binomial generalized linear model and a Benjamini-Hochberg correction was applied. Statistically significant differences between groups are shown as *$p<0.05$ and **$p<0.01$. All figures were created with GraphPad Prism (version 9.3.0, GraphPad Software, San Diego, California, USA). VEG = vegetable oil, MILK = bovine milk fat, NBW = normal birth weight, IUGR = intrauterine growth restricted.
